# Th17 Cytokines Disrupt the Airway Mucosal Barrier in Chronic Rhinosinusitis

**DOI:** 10.1155/2016/9798206

**Published:** 2016-01-19

**Authors:** Mahnaz Ramezanpour, Sophia Moraitis, Jason L. P. Smith, P. J. Wormald, Sarah Vreugde

**Affiliations:** ^1^Department of Surgery (Otorhinolaryngology Head and Neck Surgery), The Queen Elizabeth Hospital and The University of Adelaide, Adelaide, SA 5011, Australia; ^2^School of Biology, Faculty of Science and Engineering, Flinders University of South Australia, Adelaide, SA 5042, Australia

## Abstract

Cytokine mediated changes in paracellular permeability contribute to a multitude of pathological conditions including chronic rhinosinusitis (CRS). The purpose of this study was to investigate the effect of interferons and of Th1, Th2, and Th17 cytokines on respiratory epithelium barrier function. Cytokines and interferons were applied to the basolateral side of air-liquid interface (ALI) cultures of primary human nasal epithelial cells (HNECs) from CRS with nasal polyp patients. Transepithelial electrical resistance (TEER) and permeability of FITC-conjugated dextrans were measured over time. Additionally, the expression of the tight junction protein Zona Occludens-1 (ZO-1) was examined via immunofluorescence. Data was analysed using ANOVA, followed by Tukey HSD post hoc test. Our results showed that application of interferons and of Th1 or Th2 cytokines did not affect the mucosal barrier function. In contrast, the Th17 cytokines IL-17, IL-22, and IL-26 showed a significant disruption of the epithelial barrier, evidenced by a loss of TEER, increased paracellular permeability of FITC-dextrans, and discontinuous ZO-1 immunolocalisation. These results indicate that Th17 cytokines may contribute to the development of CRSwNP by promoting a leaky mucosal barrier.

## 1. Introduction 

Chronic rhinosinusitis (CRS) is characterized by severe inflammation of the sinus mucosa leading to blockage of the nasal passageway and the accumulation of mucus and pathogens in the nose and paranasal sinuses [1, 2]. CRS affects around 1.9 million Australians [3] and puts a large financial burden on health care systems [4].

CRS is subdivided in CRS with nasal polyps (CRSwNP) and CRS without nasal polyps (CRSsNP) based on the presence or absence of polyps in the sinonasal cavities [5]. CRSwNP patients typically display a T helper 2 (Th2) polarization, whereas patients without nasal polyps (CRSsNP) are often characterized by a Th1 polarization with high levels of Interferon-*γ* [6].

Cytokines regulate innate and acquired immunity [7] and can disrupt mucosal barrier function by altering tight junction (TJ) composition and structure. This occurs through signalling pathways independent of cell death and the effect is cell type specific, pleiotropic, and time and dose-dependent [8]. Relatively few studies have demonstrated cytokine effects on nasal epithelial tissue or barrier function [5, 6, 9, 10]. Th1 cytokines such as interleukin-2 (IL-2), interferon-*γ* (IFN-*γ*), and Tumour Necrosis Factor alpha (TNF-*α*) are the primary source for proinflammatory Th1 responses [11], in which they are effective in controlling infection with intracellular pathogens and for perpetuating autoimmune responses [12]. In contrast, Th2 immune responses are characterized by the production of the interleukins IL-4, IL-5, and IL-13 [13] that are associated with the promotion of eosinophil recruitment and activation, and inhibition of several macrophage functions, thus providing phagocyte-independent protective responses [14]. Th17 cells are a subset of activated CD4^+^ T cells and are characterized by the production of the interleukins IL-17A, IL-17F, IL-22, and IL-26 [15]. Th17 cells act as a bridge between adaptive and innate immunity where they play crucial roles in the development of autoimmunity, inflammation, and allergic reactions [10]. Here, we tested the effect of interferon proteins and of Th1, Th2, and Th17 cytokines on the mucosal barrier structure and function of primary nasal epithelial cells harvested from nasal polyps of CRS patients.

## 2. Methods

### 2.1. Harvesting and Culturing Human Nasal Epithelial Cells* In Vitro*

Ethical approval for nasal brushing from CRS patients was granted from the Queen Elizabeth Hospital Human Ethics Committee and only consented patients were included in the study. Exclusion criteria included active smoking, age less than 18 years, and systemic disease. Primary human nasal epithelial cells (HNECs) were harvested from nasal polyps by gentle brushing in a method as described in [16]. Extracted cells were suspended in Bronchial Epithelial Growth Media (BEGM, CC-3170, Lonza, Walkersvill, MD, USA), supplemented with 2% Ultroser G (Pall Corporation, Port Washington, NY, USA). The cell suspension was depleted of monocytes using anti-CD68 (Dako, Glostrup, Denmark) coated culture dishes, and HNECs expanded in routine cell culture conditions of 37°C humidified air with 5% CO_2_ in collagen coated flasks (Thermo Scientific, Walthman, MA, USA). HNECs were tested at passage two and confirmed to be of epithelial lineage via reactivity to pan-Cytokeratin and CD45 antibodies (both from Abcam, Cambridge, MA, USA), and a Diff-Quick staining method used in the assessment of cell morphology by professional cytologists (IMVS, The Queen Elizabeth Hospital, Woodville, Australia).

### 2.2. Air Liquid Interface Culture

HNEC were maintained at an Air Liquid Interface (ALI) medium, following the Lonza ALI culture method (Lonza, Walkersville, USA). Briefly, Transwells (BD Biosciences, San Jose, California, USA) were treated with collagen (Stemcell Technologies, Australia). 70,000 cells were seeded in a volume of 100 *μ*L B-ALI medium into the apical chamber of the Transwell plate and 500 *μ*L of B-ALI growth medium was added to the basal chamber in all wells containing the inserts. Cells were incubated at 37°C. On day 3 after seeding, B-ALI growth medium was removed from the apical and basal chambers and 500 *μ*L B-ALI differentiation medium was added to the basal chamber only, exposing the apical cell surface to the atmosphere. Regular examinations were made to assess the integrity of the cell layer.

### 2.3. Th1, Th2, and Th17 Cytokines Exposure

Cytokines were added to the basal Transwell chamber at the following final concentrations: recombinant human Interferon-*γ* (500 ng/mL, Sigma, Saint Louis, USA), interferon *β* 1a (50 ng/mL, Sigma, Saint Louis, USA), interferon-*α* (500 ng/mL, Sigma, Saint Louis, USA), Tumour Necrosis Factor-*α* (500 ng/mL, Sigma, Saint Louis, USA), IL-1b (500 ng/mL, Sigma, Saint Louis, USA), IL-4 (50 ng/mL, Gibco, life Technology, USA), IL-5 (50 ng/mL, Gibco, Life Technology, USA), IL-13 (50 ng/mL, Gibco, Life Technology, USA), IL-17A (50 ng/mL, Gibco, Life Technology, USA), recombinant human IL-22 (50 ng/mL, Sigma, Saint Louis, USA), and recombinant human IL-26 (50 ng/mL, Abnova Taiwan Corp).

### 2.4. Hematoxylin and Eosin (H&E) Staining and Histopathological Examination of Slides

Paraffin-embedded tissue samples were cut in 4 *μ*m thick sections on a microtome (Thermo Scientific HM 325 Rotary Microtome). Slides were then stained with routine hematoxylin and eosin (H&E) staining using Mayer's Hematoxylin and Eosin (Lillie's Modification, Dako, Thermo Fisher Scientific, Waltham, MA, USA). All slides were then scanned using digital whole-slide imaging technology (WSI) on the NanoZoomer Digital Pathology System (Hamamatsu Photonics, Hamamatsu City, Japan) under high resolution (40x objective magnification power). Eosinophil and neutrophil scoring was done according to a systematized methodology as detailed in [17].

### 2.5. Transepithelial Electrical Resistance (TEER)

Transepithelial electrical resistance (TEER) was measured by using an EVOM volt-ohmmeter (World Precision Instruments, Sarasota, FL, USA). Briefly, 100 *μ*L of B-ALI medium was added to the apical chamber of ALI cultures to form an electrical circuit across the cell monolayer and into the basal chamber. Cultures were maintained at 37°C during the measurement period using a heating platform. Only wells displaying baseline resistance readings greater than 500 Ω/cm^2^ were used for the experiments. Cytokines and control (B-ALI medium for the negative control and 2% Triton ×100 for the positive control) were added to the bottom chamber of each well, and TEER measurements were obtained at time 0, 4 h, and 24 h.

### 2.6. Permeability Assay

Paracellular permeability was studied by measuring the apical-to-basolateral flux of FITC dextran 4 kDa (Sigma, Saint Louis, USA). Briefly, after treating the cells for 24 h, the upper chambers were filled with 3 mg/mL of FITC-dextran and incubated for 2 h at 37°C. 40 *μ*L samples were recovered from the bottom chamber and serially diluted on a 96-well plate (Corning Costar cell culture plates (96 wells)), and the fluorescence was measured with a microplate fluorometer (FLUOstar Optima, BMG Labtech, Ortenberg, Germany).

### 2.7. Cytotoxicity Assay

The amount of lactate dehydrogenase (LDH) in the medium was measured at 24 hours using the Cytotox Homogeneous Membrane Integrity Assay (Promega, Australia). Briefly, 50 mL of the media from each well was transferred to a new plate, and 50 mL of LDH reagent was added to the supernatant and incubated for 30 minutes in the dark at room temperature. The OD was measured at 490 nm on a FLUOstar OPTIMA plate reader (BMG Labtech, Ortenberg, Germany).

### 2.8. Trypan Blue Assay

After cell incubation with the cytokines, the culture medium was discarded and washed with PBS, and 100 *μ*L of trypsin was added to each well. The plate was incubated for 5 minutes, and then 250 *μ*L of supplemented culture medium was added. The contents of each well were aspirated, placed into labeled microtubes, and centrifuged at 1000 rpm for 5 minutes. The supernatant was discarded, and the cells were suspended again in 100 *μ*L of culture medium. An aliquot of 10 *μ*L of cell suspension was removed and mixed with 10 *μ*L of Trypan Blue. After homogenization, the live and dead cells were counted and the percentage of viable cells was calculated.

### 2.9. Immunofluorescence Microscopy

Cells were fixed with 2.5% formalin in phosphate-buffered saline (PBS) for 10 min, and then the cells were rinsed with tris-buffered saline-0.5% Tween (TBST) four times, permeabilized with 1% SDS in PBS, and blocked with serum free blocker (SFB; Dako, Glostrup, Denmark) for 60 minutes, at room temperature. Mouse monoclonal anti-human ZO-1 (Invitrogen, Carlsbad, CA, USA), diluted to 5 *μ*g/mL in TBST-10% SFB, was added to the excised culture support membranes and allowed to incubate for 1 hour at room temperature. Excess primary antibody was removed with TBST, and 2 *μ*g/mL anti-mouse Alexa-594 conjugated secondary antibody (Jackson ImmunoResearch Labs Inc., West Grove, PA, USA) was then added for 1 hour at room temperature. The filters were rinsed in TBST, and after the third wash 200 ng/mL of 4′,6-diamidino-2-phenylindole (DAPI; Sigma, Aldrich) was added to resolve nuclei. Membranes were rinsed once with ultrapure water, and 95% ice cold ethanol was added for 1 hour at 4°C. Membranes were transferred to a glass slide and a drop of anti-fade mounting medium (Dako, Glostrup, Denmark) was added before cover-slipping. Samples were visualized by using a LSM700 confocal laser scanning microscope (Zeiss Microscopy, Germany).

### 2.10. Statistical Analysis

Data are presented as mean ± SEM. The TEER experiment was performed using three replicates from four CRSwNP patients with values normalised against the mean value from the patient at time 0. The dextran-FITC assay was performed using three replicates from four CRSwNP patients. Statistical analyses of all data were carried out using ANOVA, followed by Tukey HSD post hoc test. These tests were performed using SPSS software (version 18).

## 3. Results

### 3.1. Th17 Cytokines Disrupt TEER of HNECs

**Figure 1:**
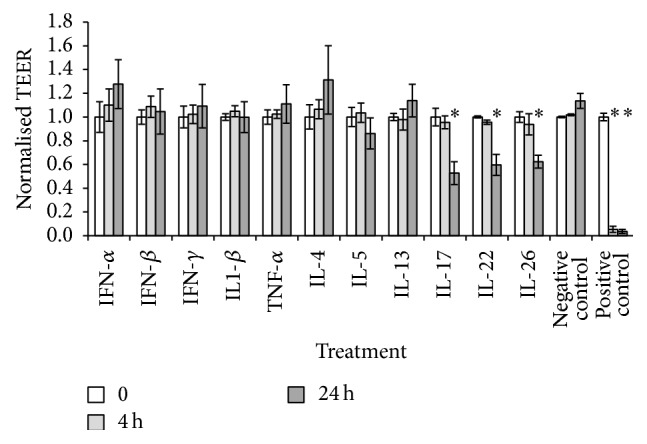
The Th17 cytokines IL-17, IL-22, and IL-26 cause a time-dependent reduction in TEER. Effect of Interferons *α*, *β*, and *γ* and of Th1, Th2, and Th17 cytokines on epithelial barrier function determined by measuring transepithelial electrical resistance (TEER) before the addition of cytokines (*t* = 0), and after 4 and 24 h. The values are shown as mean ± SEM for *n* = 4. Treatments significantly different from the untreated control at *p* < 0.05 are presented as *∗*. ANOVA, followed by Tukey HSD post hoc test.

ALI cultures were established from 4 independent CRS patient donors (2 males and 2 females, aged 45–60 years). Two patients were diagnosed with grass-pollen allergy, one had Aspirin-Exacerbated Respiratory Disease (AERD), and two were asthmatic. Eosinophil and neutrophil counts [11.1 (4.6–21.3) and 0.8 (0–2.4)] per High Power Field (HPF) were not different between the different patients (*p* > 0.05). The effect of interferons and of Th1, Th2, and Th17 cytokines was examined by measuring the TEER across HNEC monolayers from CRS patients at different time points. All Th17 cytokines tested (IL-17, IL-22 and IL-26) caused a significant reduction in TEER (average of 1.9 times; 1.7 times; 1.61 times for IL-17, IL-22, and IL-26 resp.) after 24 h of incubation. In contrast, Th1 and Th2 cytokines or interferon *α*, *β*, or *γ* did not show any significant effect on TEER (Figure 1).

### 3.2. Th17 Cytokines Increase the Paracellular Permeability of HNECs

**Figure 2:**
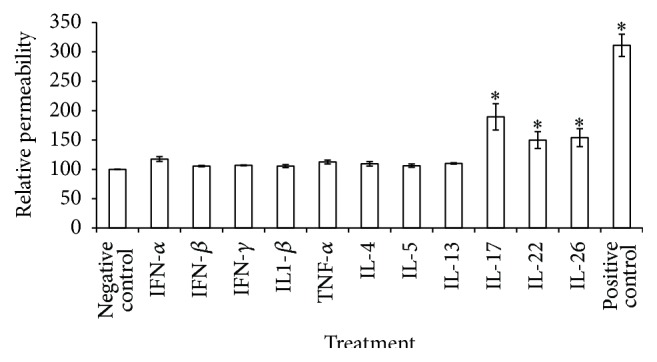
The Th17 cytokines cause an increase in paracellular permeability in CRS. Paracellular permeability of HNECs in ALI culture was determined by a Dextran-FITC assay after 24 h treatment of Th1, Th2, and Th17 cytokines. The values are shown as means ± SEM for *n* = 4. Treatments significantly different from the untreated control at *p* < 0.05 are presented as *∗*. ANOVA, followed by Tukey HSD post hoc test.

All IL-17 family cytokines (IL-17, IL-22, and IL-26) led to a significant enhancement of paracellular permeability (*p* < 0.05) (Figure 2). IL-17 had the strongest effect, with 89.33% of the fluorescent dextran crossing the HNEC monolayer whereas IL-22 and IL-26 increased paracellular permeability with 49.85% and 53.92%, respectively. Th1 and Th2 cytokines and interferons *α*, *β*, and *γ* did not show any significant effect on the paracellular permeability in CRS patients (Figure 2).

### 3.3. Tight Junction Disruption Does Not Correlate with Cytotoxicity

**Figure 3:**
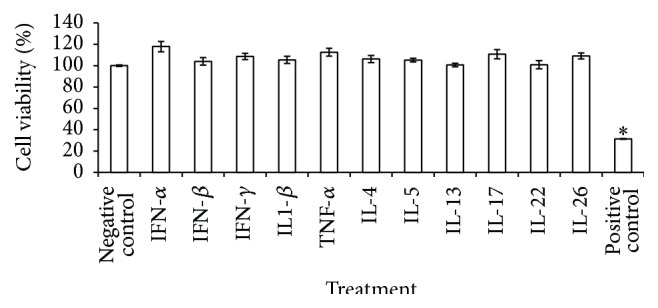
Th1, Th2, and Th17 cytokines and interferons do not have cytotoxic effects on HNECs. Relative viability as determined by the LDH assay after 24 h treatment of Th1, Th2, and Th17 on HNECs. Cell viability was calculated relative to the untreated cells (0 *μ*g/mL) as negative control. The values are shown as means ± SEM, *n* = 4.

The effect of interferons and of Th1, Th2, and Th17 cytokines on cellular toxicity was examined by measuring LDH release from HNECs. There was no statistically significant increase in LDH release after 24 h incubation with any of the cytokines (Figure 3). In addition, the cell density estimated by the Trypan Blue assay did not show any significant differences in cell density or cell viability in cytokine treated cells compared to control cells (results not shown).

### 3.4. Th17 Cytokines Cause a Profound Disruption of the Tight Junction Protein ZO-1

**Figure 4:**
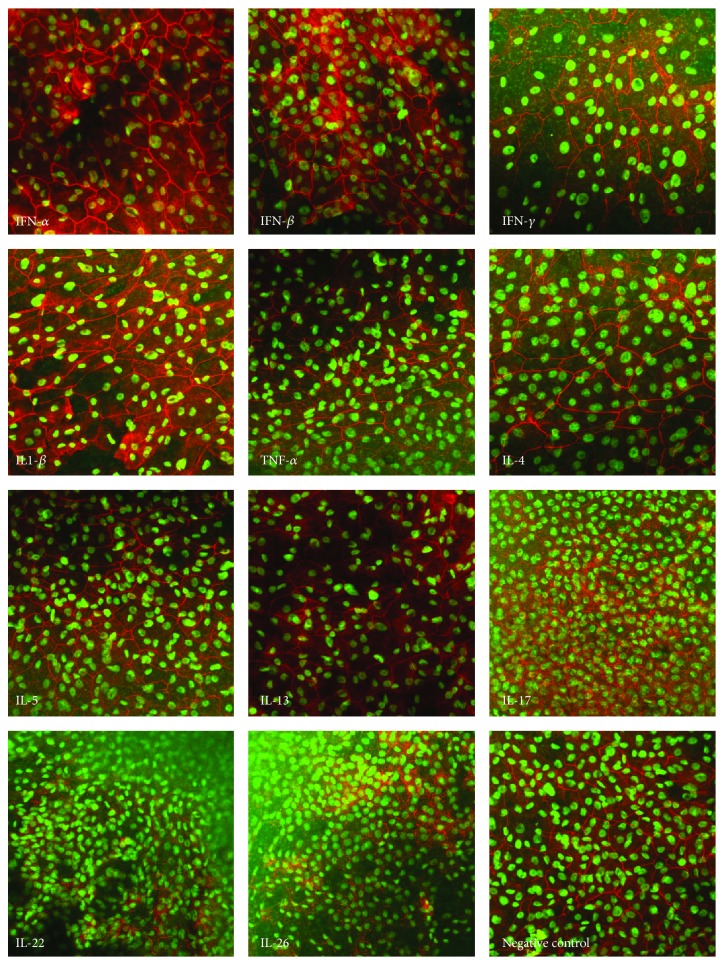
The Th17 cytokines cause a profound disruption of the tight junction in CRS. Effect of IFN-*α*, IFN-*β*, IFN-*γ*, IL-1*β*, TNF-*α*, IL-4, IL-5, IL-13, IL-17, IL-22, IL-26, and negative control on the localization of Zona Occludens-1 (ZO-1) (in red with DAPI stain in green) was imaged with 20x objective power using immunofluorescence confocal laser scanning microscopy 24 hours after application of the cytokines.

The effect of interferon proteins and of Th1, Th2, and Th17 cytokines on the localization of Zona Occludens-1 (ZO-1) was examined by using immunofluorescence staining and confocal laser scanning microscopy, 24 hours after application of the cytokines. In untreated cells, ZO-1 was located at the periphery of the apical side of the monolayer, as expected. Similarly, interferons *α*, *β*, and *γ* and Th1 and Th2 cytokines, which had no effect on either TEER or paracellular permeability, led to no alterations in the localization of ZO-1. In contrast, application of Th17 cytokines, which significantly altered epithelial barrier function, resulted in profound disruption of ZO-1 immunolocalisation evidenced by faint or discontinuous regions of fluorescence (Figure 4).

## 4. Discussion

Cytokine mediated insult on mucosal membranes, causing disruption of tight junctions and increased paracellular permeability, contributes to a multitude of pathologic conditions in inflammatory diseases of the upper airways [18–20]. In this study, we compared the effect of interferons and of signature Th1, Th2, and Th17 cytokines on the barrier function of primary nasal epithelial cells harvested from CRS patients with nasal polyps. Immunolocalisation of the tight junction protein ZO-1 was used to analyse tight junction integrity to gain insights into mechanisms of cytokines dependent disruption of the airway epithelial barrier. Our study indicates that, in CRSwNP patients, IL-17 family cytokines (IL-17A, IL-22, and IL-26) can significantly disrupt epithelial barrier function in association with a disruption of tight junction integrity and without causing cellular toxicity. In contrast, Th1 and Th2 cytokines or interferons showed no significant difference on either TEER or paracellular permeability of HNECs.

It has been well established that different cytokines cause different, often opposing effects on epithelial barrier function depending on the cell type used and that any observed effect is dose and time-dependent (reviewed in [8]). Our results indicate that application of Th1 cytokines such as IFN-*γ* and TNF-*α* does not have detrimental effects on epithelial barrier function. Rather, application of these cytokines appeared to slightly enhance the TEER of human nasal epithelial monolayers derived from some of the CRSwNP patients 24 hours after application. Whereas IFN-*γ* and TNF-*α* generally decrease barrier function in different cell lines [21, 22], in airway epithelial cells, IFN-*γ* has been reported to decrease barrier function by Soyka et al. [5] and promote epithelial barrier function by Ahdieh et al. [23]. These differences in the response to IFN-*γ* could be attributed to many factors including interindividual variability in response to cytokines, different origin of cells (mucosa or polyps), and differences in experimental techniques. In the experiments by Soyka et al. [5], for example, TEER changes in response to IFN-*γ* treatment from CRSwNP, CRSsNP and controls were pooled while in our studies, only cells from CRSwNP were used. Given the small number of samples used in most studies, further experiments using a larger number of donors will need to address the cause of these discrepancies. TNF-*α* can increase TEER in mammalian uterine cell monolayers in a dose-dependent manner [24]. Interestingly, a recent study revealed that patients had developed a recurrence of CRS after the start of TNF-*α* inhibitor administration with a remission of the disease only after cessation of TNF-*α* inhibitor treatment [25].

Moreover, we demonstrated no significant reduction of TEER by Th2 family cytokines (IL-4, IL-5, and IL-13) after 4 h and 24 h in our experiments. Using airway epithelial cells, Saatian et al. showed that IL-4 and IL-13 caused a reduction in TEER 72 h after challenge but not after 24 h [26]. Soyka et al. [5] also used airway epithelial cells from CRSwNP patients (*n* = 2) and controls (*n* = 2) and similarly showed significantly decreased TEER after IL-4 challenge; however, this effect was already evident after 12 and 24 hours. The reason for these discrepancies is not clear and can be dependent on numerous factors. While physiologically relevant, it is well known that experiments using primary cells have limitations due to inherent interindividual differences of age, genetic make-up and medical history [27]. This is particularly important in CRS, a multifactorial disease that can be associated with Th1 or Th2 responses. Also ethnicity may play a role as Caucasian CRSwNP patients are often characterised by a predominant Th2 type eosinophilic inflammation with high level of IL-5, whereas Asian CRSwNP patients preferentially have a Th1/Th17 polarization signature [28].

Th17 cells play a significant role in chronic allergic airway inflammation [29]. Th17 cytokines also affect the gut mucosal barrier function by promoting the amplification of the host response to secrete neutrophil chemoattractants and antimicrobial peptides such as lipocalin-2 and calprotectin. In addition, Th17 cells can expand within mucous layers in association with the presence of pathogens that are resistant to some of the induced antimicrobial responses [30]. In allergic rhinitis, serum IL-17 levels were significantly related to clinical severity [31]. The expression level of IL-17 was also shown to be significantly higher in recalcitrant CRSwNP compared to controls [32]. In the present study, we found that IL-17 induced barrier dysfunction as assessed by reduction in TEER and enhanced macromolecular permeability, whereas Soyka et al. showed that IL-17 had no influence on TEER [5]. We also demonstrated significant reductions in TEER and enhanced macromolecular permeability of IL-22 and IL-26 which is the first such analysis using human nasal epithelial cells.

In tight junction formation, ZO-1 plays an essential role, by linking the transmembrane proteins occludin, claudin, and Junction Adhesion Molecule (JAM) cytoplasmic components of the tight junctions to the actin cytoskeleton [33]. Disruption of the actin-myosin structure has been understood to modulate paracellular permeability [8]. We observed a loss of normal ZO-1 immunolocalisation in HNEC monolayers of CRSwNP patients secondary to challenge with Th17 (IL-17, IL-22, and IL-26) cytokines, in association with disruption of barrier function. We also observed that IL-17, IL-22, and IL-26 treated cells appeared in higher cell densities than cells treated with other Th1 or Th2 family cytokines. It is known that TJs regulate epithelial proliferation by different molecular mechanisms, which generally suppress proliferation as cell density (and hence TJ assembly) increases (reviewed in [34]). Changes in expression of ZO-1 and ZO-1-associated nucleic acid binding protein (ZONAB), a Y-box transcription factor, affect cell proliferation; however, these effects take place at least 48 hours after changes in gene expression [35]. Given that the duration of exposure to the treatments in our experiments was only 24 hours and that cell counts did not show significant differences, we believe that TJ disruption secondary to IL-17, IL-22, and IL-26 exposure might render the pseudostratified layer of cells into a monolayer which might appear relatively overcrowded.

In summary, in patients with CRSwNP, Th1 and Th2 cytokines showed no significant effect on epithelial barrier structure and function. In contrast, the Th17 cytokines family (IL-17, IL-22, and IL-26) showed significant disruption of the epithelial barrier, leading to increased paracellular permeability associated with reduced tight junctionintegrity. In future studies, it will be important to determine the cellular mechanism of the effect of Th17 cytokines on the mucosal barrier in CRS patients to provide an opportunity for therapeutic modulation in inflammatory stress.
